# Clinical characteristics of *Mycoplasma pneumoniae* pneumonia with airway involvement in children

**DOI:** 10.1002/ped4.12457

**Published:** 2025-01-06

**Authors:** Baoying Zheng, Yuchun Yan, Ling Cao

**Affiliations:** ^1^ Department of Pulmonology The Children's Hospital Affiliated to the Capital Institute of Pediatrics Beijing China; ^2^ Department of Radiology The Children's Hospital Affiliated to the Capital Institute of Pediatrics Beijing China

**Keywords:** Children, *Mycoplasma pneumoniae*, Community‐acquired pneumonia, Bronchiolitis obliterans

## Abstract

**Importance:**

*Mycoplasma pneumoniae* pneumonia (MPP) with chest computed tomography (CT) findings showing airway involvement as the main manifestation has begun to be noted and increasingly reported. This type of MPP has different clinical features and may progress to bronchiolitis obliterans (BO). Early recognition and treatment are helpful for reducing sequelae.

**Objective:**

To investigate the clinical characteristics of MPP patients with airway involvement and provide guidance for clinical recognition of this type.

**Methods:**

Data from children diagnosed with MPP were collected. Forty‐one patients were assigned to the airway group according to chest CT, and 114 patients were assigned to the air space group. The clinical data of the two groups were compared and analyzed.

**Results:**

The children in the airway group were younger, and the prevalence of wheezing, pulmonary moist rales, and allergic background in the airway group was greater. The prevalence of severe MPP, the proportions of neutrophils, C‐reactive protein, and D‐dimer were lower in the airway group than in the air space group. Significantly more patients had lung involvement in both airways in the airway group. No cases of BO were found in the airway group.

**Interpretation:**

*Mycoplasma pneumoniae*‐associated airway involvement mostly occurs in young children, especially in atopic individuals. Patients with this type of pneumonia are prone to have clinical wheezing and pulmonary moist rales. The airway group included relatively few severe cases, but more patients had involvement of both lungs. Whether the patients in the airway group had a greater chance of developing BO needs further investigation.

## Graphical abstract

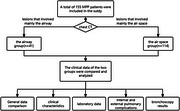

## INTRODUCTION

*Mycoplasma pneumoniae* (MP) is one of the most important pathogens responsible for community‐acquired pneumonia (CAP) in children. The prevalence of *Mycoplasma pneumoniae* pneumonia (MPP) can reach 10%–40% in children hospitalized with CAP.^1^ In addition to infections of the respiratory system, MP contributes to a broad array of extrapulmonary diseases, such as rashes, reactive arthritis, and gastrointestinal, nervous, and hematological system diseases.^2^, ^3^ Severe MPP (SMPP) can present as residual sequelae such as atelectasis, bronchiectasis, and bronchiolitis obliterans (BO).^4^

MPP has various imaging manifestations. Chest computed tomography (CT) clearly reveals lung parenchymal and interstitial lesions, which are closely related to pathological findings and are currently widely used in clinical practice. Among the chest CT findings, consolidation, in which the air space is mainly involved, is the most common type. In recent years, MPP with airway involvement as the main manifestation has been increasingly reported, which has been called MPP bronchiolitis in some studies and is thought to have the potential to progress to BO.^5^ At present, few studies have focused on the clinical manifestations and outcomes of MPP with airway involvement. This study summarized and analyzed cases of MP‐associated airway involvement to enhance the understanding of the disease so that pediatricians can identify this type of MPP early and reduce the occurrence of sequelae in children.

## METHODS

### Ethical approval

This study was approved by the Ethics Committee of The Children's Hospital Affiliated to the Capital Institute of Pediatrics (SHERLL2021010). This study was a retrospective study and the data were anonymous, therefore the requirement for informed consent was waived.

### Study population

Among the children diagnosed with MPP who were admitted to the respiratory department of The Children's Hospital Affiliated with the Capital Institute of Pediatrics from February 2021 to February 2022, 41 patients whose chest CT scans showed lesions that involved mainly the airway were assigned to the airway group, whereas 114 patients whose chest CT scans showed lesions that involved mainly the air space were assigned to the air space group.

### Diagnostic criteria

MPP diagnosis: Patients met the criteria for pneumonia and MP infection. A diagnosis of pneumonia was made as follows: ^6^ acute respiratory infection symptoms (fever, cough, or wheezing) upon physical examination and chest imaging with infiltrates. MP infection was confirmed by conducting a serological test (MP‐IgM‐positive and an antibody titer ≥ 1:160 or a fourfold or greater increase in the titer), and MP nucleic acid was detected in the nasopharyngeal aspirate and/or alveolar lavage fluid (Dual Amplification kits; Wuhan Zhongzhi Biotechnology Co., Ltd.).^7^ The MP‐IgM was detected by colloidal gold kits (Beijing Bell Bio‐engineering Co., Ltd.). The antibody titer of MP was detected by Passive Particle Agglutination kits (SERODIA‐MYCO II; Zhuhai Lizhu Reagent Co., Ltd.).

SMPP was defined as an MPP plus one of the following characteristics^8^: 1) a poor general condition; 2) an increased respiratory rate (infants > 70 breaths/min and older children > 50 breaths/min); 3) dyspnea and cyanosis; and 4) multilobe invasion or involvement of ≥ 2/3 of the lung; 5) extrapulmonary complications; or 6) pleural effusion, and 7) transcutaneous oxygen saturation in room air ≤ 92%.

The airway group and air space groups were classified according to chest CT findings. The airway group was defined on the basis of a chest CT examination showing a predominant unilateral or bilateral tree‐in‐bud pattern, with centrilobular nodules and bronchiolar wall thickening^9^ representing pathological small caliber airway involvement pathologically. The air space group was defined on the basis of a chest CT examination showing predominant homogenous consolidation in at least one lobe/segment, with or without an air bronchogram in the lesion,^10^ which represented pathological air space involvement.

### Inclusion and exclusion criteria

The inclusion criteria were that all patients met the diagnostic criteria for MPP. The chest CT images with the most severe manifestations were obtained and classified by a radiologist with 20 years of working experience. For patients with multiple findings on chest imaging, the most dominant finding was chosen. A total of 41 patients were classified into the airway group, and 114 patients were classified into the air space group.

The exclusion criteria for patients were as follows: 1) Evidence of coinfection with other pathogens was obtained within 7 days after admission, and etiological tests included cultures for bacteria and fungi and viral nucleic acid detection. Dual amplification kits (Wuhan Zhongzhi Biotechnology Co., Ltd.) were used for the detection of respiratory syncytial virus, adenovirus, metapneumovirus, influenza, and parainfluenza; 2) Patients had a disease course ≥ 2 weeks at admission or a normal temperature for more than 1 week; 3) used glucocorticoids for more than 3 days before admission; and 4) had chronic lung disease, congenital heart disease, immunodeficiency, or heredity neurological disorders.

### Data collection

Clinical information was retrospectively collected from the medical records, which included: age, sex, clinical symptoms and signs, intrapulmonary and extrapulmonary complications, fever duration, the maximum levels of the laboratory tests observed during hospitalization, including white blood cell (WBC) counts, neutrophil counts, C‐reactive protein (CRP), lactate dehydrogenase (LDH), D‐dimer and the serum total immunoglobulin E (IgE) concentrations. Patients with more than 2/3 high‐density uniform consolidation of a single lobe (indicating the presence of mucous embolus or plastic bronchitis) and those with diffuse broncho‐bronchiolitis radiographic indications and clinical manifestations of wheezing and dyspnea (indicating mucous embolus blockage) underwent fiber bronchoscopy with bronchoalveolar lavage.^11^ Plain chest radiographs and chest CT were performed at the follow‐up visit. An electrocardiogram, echocardiography, and abdominal ultrasound were performed on all patients.

### Allergic background

Patients with any of the following 3 conditions were identified as having an allergic background: 1) personal history of allergic diseases such as asthma, allergic rhinitis, atopic dermatitis, and food allergy; 2) one or more positive allergen serum‐specific IgEs; and 3) a positive skin prick test.^12^

### Statistical analysis

The statistical analyses were performed via SPSS software (IBM, version 22). Normally distributed data are reported as the means ± standard deviations. Data with a skewed distribution are presented as median values (interquartile ranges: Q1, Q3). The different groups were compared using a two‐sample *t*‐test or the Mann‒Whitney *U* test. The statistical significance of the differences in the categorical variables was determined using the chi‐square test. Statistical significance was determined at the two‐tailed 0.05 level.

## RESULTS

### General data comparison

Forty‐one patients were included in the airway group, 23 males and 18 females, and the mean age was 5.8 ± 3.0 years. One hundred fourteen patients were included in the air space group, 54 males and 60 females, with an average age of 6.8 ± 2.5 years. No significant difference in sex was observed between the two groups (*χ* ^2^ = 0.919, *P* = 0.338), and the children in the airway group were younger than those in the air space group (*t* = 1.988, *P* = 0.049).

### Comparison of clinical characteristics

The proportions of patients with wheezing, pulmonary moist rales, and an allergic background in the airway group were greater than those in the air space group. The proportion of SMPP patients was lower than that in the air space group (Table 1).

**TABLE 1:** Comparison of clinical features between the two groups

Clinical index	Airway group (*n* = 41)	Air space group (*n* = 114)	*Z*/*χ* ^2^	*P‐*value
Fever duration (days)	9.0 (7.5, 11.0)	10.0 (8.0, 11.3)	−0.612	0.540
Wheeze	10 (24.4)	8 (7.0)	7.255	0.007
Moist rales	26 (63.4)	50 (43.9)	4.614	0.032
Allergic background	18 (44.0)	25 (21.9)	7.263	0.007
SMPP	9 (22.0)	45 (39.5)	4.078	0.043

Data are shown as median (Q1, Q3) or *n* (%).

Abbreviation: SMPP, severe *Mycoplasma pneumoniae* pneumonia.

### Comparison of laboratory data

The proportions of neutrophils and the levels of D‐dimer in the airway group were lower than those in the air space group, and the levels of CRP levels were significantly lower than those in the air space group. Moreover, the WBC counts, LDH levels, and serum total IgE levels did not differ significantly between the two groups (Table 2).

**TABLE 2:** Comparison of the laboratory data between the two groups

Laboratory data	Airway group (*n* = 41)	Air space group (*n* = 114)	*Z*/*t*	*P‐*value
WBC count (×10^9^/L)	8.78 (7.31, 11.39)	8.86 (7.36, 11.32)	−0.193	0.847
Neutrophil (%)	62.47 ± 10.95	67.35 ± 9.90	2.629	0.009
CRP (mg/L)	11.93 (6.15, 24.10)	26.05 (12.81, 43.45)	−3.730	<0.001
D‐dimer (mg/L FEU)	0.56 (0.35, 0.98)	1.00 (0.55, 2.22)	−3.345	0.001
LDH (U/L)	300.00 (254.50, 342.00)	309.50 (267.75, 379.75)	−1.736	0.082
Serum total IgE (IU/mL)	102.95 (42.90, 487.25)	117.00 (39.75, 369.25)	−0.278	0.781

Data are shown as median (Q1, Q3) or mean ± standard deviation.

Abbreviations: CRP, C‐reactive protein; FEU, fibrinogen equivalent unit; LDH, lactate dehydrogenase; WBC, white blood cells.

### Comparison of intrapulmonary and extrapulmonary complications

Seventeen patients (41.5%) in the airway group had complications, and 50 patients (43.9%) in the air space group developed complications; no significant difference was observed between the two groups (*χ* ^2^ = 0.071, *P* = 0.791). A detailed description of the complications is provided in Table 3.

**TABLE 3:** Complications of the two groups [*n* (%)]

Complications	Airway group (*n* = 41)	Air space group (*n* = 114)
Hypoxemia or respiratory failure	7 (17.1)	9 (7.9)
Rash	2 (4.9)	7 (6.1)
Digestive system	6 (14.6)	25 (21.9)
Cardiovascular system	2 (4.9)	10 (8.8)
Hematologic system	1 (2.4)	3 (2.6)
Pleural effusion	1 (2.4)	18 (15.8)
Atelectasis	0	11 (9.6)
Pulmonary necrosis	0	2 (1.8)
Plastic bronchitis	0	4 (3.5)

### Bronchoscopy results

In the airway group, 34 of the 41 patients underwent bronchoscopy. Among them, nine patients (26.5%) exhibited a large amount of secretion under bronchoscopy, accompanied by airway congestion. In the air space group, 102 of the 114 patients underwent bronchoscopy. Thirty‐three patients (32.4%) had large amounts of secretions with airway congestion under bronchoscopy, and the proportion of large secretions did not differ significantly between the two groups (*χ* ^2^ = 0.413, *P* = 0.520).

### Chest imaging results of the two groups

Chest CT revealed bilateral lesions in 22 patients (53.7%) in the airway group. Twenty‐three patients (20.2%) presented bilateral lesions in the air space group, and significant differences were observed between the two groups (*χ* ^2^ = 16.408, *P* < 0.001). Nine patients were diagnosed with SMPP in the airway group, and 8 patients had diffuse lesions in both lungs. In the airway group, 35 patients (85.4%) returned to our outpatient clinic within 1–3 months of the diagnosis of the disease. Six patients had plain chest radiographs, and no abnormal information was observed. Chest CT was performed for the remaining 29 patients, 27 (93.1%) of whom experienced an obvious improvement in their lung lesions. In the air space group, 97 patients (85.1%) returned to the outpatient clinic 2 weeks, 1 month, and 3 months after discharge. Among them, plain chest radiographs were obtained for 23 patients, 74 patients underwent chest CT, and 90 patients (92.8%) experienced obvious improvement. No cases of BO were identified in either group.

## DISCUSSION

Consolidation is the most common imaging manifestation of MPP. However, in recent years, MP‐associated airway involvement has begun to be noted by pediatricians, showing a unilateral or bilateral tree‐in‐bud pattern, centrilobular nodules, and bronchiolar wall thickening which represent pathological small‐caliber airway involvement. Some scholars refer to this condition as MP‐associated bronchiolitis. Bronchiolitis caused by MP infection accounts for 5%–11.5% of the total infection types.^13^, ^14^, ^15^ This type has different clinical characteristics, and because of the small caliber airway involvement, a greater chance of developing BO may exist. Understanding the clinical characteristics of such diseases is helpful for helping clinicians identify and treat patients in a timely manner and reduce the occurrence of sequelae. We refer to these two types of MPP as the air space group and the airway group.

In this study, compared with patients in the air space group, no significant difference in sex distribution was observed, but the airway group was younger, with an average age of 5.8 ± 3.0 years. Chen et al.^16^ reported 79 patients with MP‐induced bronchiolitis admitted from 2016 to 2018, who were mainly children aged 6 months to 12 months. Huang et al.^17^ summarized 67 patients with MP bronchiolitis with a median age of 5 years. These findings are consistent with the results of our study, suggesting that airway involvement caused by MP infection is more common in young children.

In addition, the proportions of patients with wheezing, pulmonary moist rales, and allergic backgrounds in the airway group were greater than those in the air space group. Li et al.^18^ reported that the incidence of wheezing, moist rales, and an allergic background in the MP airway group was significantly greater than that in the air space group, which was consistent with the results of our study. Considering that small airways accompany inflammatory exudation or granulation tissue formation,^19^ patients are more likely to develop moist rales and wheezing, especially those with an allergic background.

Laboratory examinations revealed that the proportions of neutrophils and the CRP and D‐dimer levels were lower in the airway group than in the air space group, and the SMPP in the airway group was lower than that in the air space group. CRP is a commonly used indicator of inflammation in clinical practice, which is closely related to the severity of MPP and can significantly increase in severe cases.^20^ As a specific marker of fibrinolysis, D‐dimer reflects the capacity to dissolve fibrin, and increasing evidence suggests that coagulation function is closely related to the inflammatory response. D‐dimer levels are often significantly elevated in severe cases and are a risk factor for pulmonary necrosis in patients with MPP.^21^ This study revealed fewer severe patients in the airway group than in the air space group, and the inflammatory reaction was less severe. Because the proportions of patients with allergic backgrounds in the airway group were greater than those in the air space group, we compared the serum total IgE levels between the two groups, but there was no significant difference between the two groups. Previous studies have shown that the total serum IgE levels are increased in children who develop MP‐related extrapulmonary diseases.^2^, ^22^, ^23^ However, our study revealed that the serum total IgE level is not correlated with the distinct imaging findings of MPP.

No significant difference in the incidence of complications was identified between the two groups, however, no cases of atelectasis, pulmonary necrosis, or plastic bronchitis occurred in the airway group. Under bronchoscopy, the proportion of patients with massive secretions in the airway group was not significantly different from that in the air space group. Additionally, this study revealed that children in the airway group exhibited a higher susceptibility to bilateral lung involvement compared to those in the air space group. Out of the nine children in the airway group diagnosed with SMPP, eight had diffuse lesions in both lungs. Therefore, clinicians should pay attention to patients with diffuse lesions in both lungs.

Some MPP cases may progress to BO, which is a serious threat to the health of children. BO refers to a chronic airflow limitation syndrome caused by a partial or complete occlusion of the official cavity after inflammation and fibrosis are initiated by various causes of bronchiolitis injury.^24^ Adenovirus is the most common cause of BO, followed by MP, which can account for 20% of cases.^25^ However, previous studies have not focused on the chest imaging manifestations in the acute phase of MPP in patients who eventually developed BO.^25^, ^26^ As MPP with airway involvement has been increasingly reported, some scholars reported that it is closely related to BO. Zhao et al.^5^ reviewed 17 cases of BO after MP infection and reported airway involvement in all patients in the acute stage. Huang et al.^17^ followed 67 patients with MPP‐related bronchiolitis, six of whom (9%) had BO. Wen et al.^9^ reviewed 71 MPP children with bronchiolitis; among these patients, six (8.5%) developed BO, and the incidence of wheezing, hypoxemia, and diffuse lesions in children who developed BO was significantly greater than in those who did not. All the above studies remind us to pay attention to the possibility of BO development in MPP patients with airway involvement. However, whether the MPP patients with airway involvement have a greater chance of progressing to BO is unclear. Huang et al.^15^ analyzed 1401 children with MPP, classified them according to chest CT, and analyzed their imaging and clinical features as well as their prognoses. Among them, three (0.6%) of the 468 patients with consolidation developed BO, and one (1.3%) of the 76 patients with airway involvement developed BO. Although the proportion was greater in the airway group, no significant difference was observed between the two groups. No cases of BO were found in this study, but the short follow‐up time prevented us from making a definitive conclusion. Therefore, the evidence to prove that MPP patients with airway involvement are more prone to develop BO than those with other imaging findings is insufficient thus far, but those with hypoxemia and diffuse lesions should be concerned and followed up closely for the early detection of BO.

In conclusion, MP‐associated airway involvement occurs mostly in young children, who are prone to clinical wheezing and moist pulmonary rales; and these patients often have an allergic background. For MPP patients with these clinical features, the possibility of MP‐associated airway involvement should be considered, and chest CT should be performed to confirm the diagnosis.

This study has several limitations. First, the number of patients in this study was small, and it was a single‐center study. Second, the follow‐up time was relatively short, limiting the ability to observe the occurrence of sequelae and preventing accurate evaluation of long‐term outcomes in children. In the future, a large‐sample long‐term follow‐up study can be conducted to reach more accurate conclusions.

## CONFLICT OF INTEREST

The authors declare no conflict of interest.
